# Reconstructing rare soil microbial genomes using *in situ* enrichments and metagenomics

**DOI:** 10.3389/fmicb.2015.00358

**Published:** 2015-04-30

**Authors:** Tom O. Delmont, A. Murat Eren, Lorrie Maccario, Emmanuel Prestat, Özcan C. Esen, Eric Pelletier, Denis Le Paslier, Pascal Simonet, Timothy M. Vogel

**Affiliations:** ^1^Environmental Microbial Genomics, Laboratoire Ampere, Centre National de la Recherche Scientifique, Ecole Centrale de Lyon, Université de LyonEcully, France; ^2^Josephine Bay Paul Center for Comparative Molecular Biology and Evolution, Marine Biological Laboratory, Woods HoleMA, USA; ^3^Commissariat à l'Energie Atomique et aux Energies Alternatives, GenoscopeEvry, France; ^4^UMR8030, Centre National de la Recherche ScientifiqueEvry, France; ^5^Université d'Evry Val d'EssonneEvry, France

**Keywords:** rare biosphere, soil, metagenomics, environmental genomics, plasmids, phages

## Abstract

Despite extensive direct sequencing efforts and advanced analytical tools, reconstructing microbial genomes from soil using metagenomics have been challenging due to the tremendous diversity and relatively uniform distribution of genomes found in this system. Here we used enrichment techniques in an attempt to decrease the complexity of a soil microbiome prior to sequencing by submitting it to a range of physical and chemical stresses in 23 separate microcosms for 4 months. The metagenomic analysis of these microcosms at the end of the treatment yielded 540 Mb of assembly using standard *de novo* assembly techniques (a total of 559,555 genes and 29,176 functions), from which we could recover novel bacterial genomes, plasmids and phages. The recovered genomes belonged to *Leifsonia* (*n* = 2), *Rhodanobacter* (*n* = 5), *Acidobacteria* (*n* = 2), *Sporolactobacillus* (*n* = 2, novel nitrogen fixing taxon), *Ktedonobacter* (*n* = 1, second representative of the family *Ktedonobacteraceae*), *Streptomyces* (*n* = 3, novel polyketide synthase modules), and *Burkholderia* (*n* = 2, includes mega-plasmids conferring mercury resistance). Assembled genomes averaged to 5.9 Mb, with relative abundances ranging from rare (<0.0001%) to relatively abundant (>0.01%) in the original soil microbiome. Furthermore, we detected them in samples collected from geographically distant locations, particularly more in temperate soils compared to samples originating from high-latitude soils and deserts. To the best of our knowledge, this study is the first successful attempt to assemble multiple bacterial genomes directly from a soil sample. Our findings demonstrate that developing pertinent enrichment conditions can stimulate environmental genomic discoveries that would have been impossible to achieve with canonical approaches that focus solely upon post-sequencing data treatment.

## Introduction

Soil microbial communities might display the highest level of bacterial diversity of any environment with a single gram reported to contain about a billion cells making up thousands to millions of different taxa (Torsvik et al., 2002; Gans et al., 2005; Roesch et al., 2007). These microorganisms harbor a large portion of Earth's biomass and are responsible for a range of critical functions including those that affect climate (Falkowski et al., 2000; Finzi et al., 2011), agriculture production (Kennedy and Smith, 1995; Giller et al., 1997), bioremediation (Galvão et al., 2005; Boubakri et al., 2006), pharmaceutical and other industrial applications (Malpartida and Hopwood, 1984; Daniel, 2004; Jacquiod et al., 2013). However, despite advances in both data generation and analysis methods during the last decade, genomic recovery from soil still represents the most critical bottleneck to develop an understanding of the evolutionary history, range of adaptation and functions of soil microorganisms.

Metagenomic approaches (the analysis of DNA recovered from the environment) have been developed to by-pass the limitations associated with cultivation efforts (Stahl et al., 1984; Handelsman et al., 1998; Rondon et al., 2000) and are now widely used to estimate the structure and functional potential of microorganisms found in soil samples (Tringe et al., 2005; Delmont et al., 2011b, 2012; Mackelprang et al., 2011; Fierer et al., 2012; Jacquiod et al., 2013; David et al., 2014; Nesme et al., 2014). Today, most soil metagenomic studies rely on taxonomic or functional annotation of short reads based on curated databases. However, the limited sensitivity of short reads restrains the fuller explanation of the available sequencing information (Wommack et al., 2008; Delmont et al., 2011a).

A more biologically promising yet computationally challenging alternative is to assemble short reads back together to reconstruct genomes and other genetic structures in a *de novo* manner. This approach was successfully conducted in various environments, including acid mine drainage biofilms (Tyson et al., 2004), sludges (Garcia Martin et al., 2006; Albertsen et al., 2013), human feces (Sharon et al., 2013), cow rumen (Hess et al., 2011), permafrost soil (Mackelprang et al., 2011), hypersaline lakes (Narasingarao et al., 2012), hot spring microbial mat (Liu et al., 2012) and hydrothermal plumes (Anantharaman et al., 2013). However, the genetic distance between the different members of the ecosystem affects the efficiency of the assembly process (Luo et al., 2012; Mende et al., 2012; Albertsen et al., 2013; Nielsen et al., 2014), leading to a drastic decline of recovery in metagenomic assemblies of complex communities.

Soil samples harbor large numbers of uniformly distributed microbial genomes, and approaches that work for other environments fail to recover genomes from soil in a systematical manner as they quickly hit the computational limitations of assembly algorithms. As a consequence, recovering even the most abundant genomes from temperate soils through metagenomic assembly approaches have yet to be accomplished (Delmont et al., 2012; Pell et al., 2012; Howe et al., 2014). For instance, Howe et al. (2014) generated 1.8 and 3.3 billion reads from two soil metagenomes, yet the length of genetic structures they could assemble remained below 21 kb and 3 kb, respectively. Today, the challenge to recover large genetic structures from a soil metagenome is unlikely to be addressed with deeper sequencing. However, employing biologically relevant modifications that take place before sequencing can reduce the complexity of challenges that arise after sequencing. For instance, we hypothesize that altering the structure of a soil microbiome in order to reduce its complexity by favoring the emergence of dominant microbial populations can improve metagenomic assemblies.

Microcosm enrichment is a common approach in bioremediation (i.e., employing microbes to degrade toxic chemical compounds) and bioprospecting (Wagner-Döbler et al., 1998; Ibekwe et al., 2001; Yakimov et al., 2005; McKew et al., 2007). Enrichment studies were also used in combination with metagenomics to recover new functions from soil (Jacquiod et al., 2013; David et al., 2014; Delmont et al., 2014) and successfully recovered novel genomes from less diverse environments (McIlroy et al., 2013). Here, we implement an enrichment-based “divide and conquer” approach in an attempt to reconstruct novel bacterial genomes directly from a soil sample collected from the Long-term Park Grass experiment, Rothamsted, UK, where previous efforts to reconstruct genomes using direct sequencing approaches failed (Delmont et al., 2012). We divided our soil sample into 23 identical microcosms and subjected them to various environmental stress conditions (ESCs), including mercury, ethanol, and diesel enrichments, in an attempt to stimulate different parts of the microbiome. After 4 months of treatment, we extracted, sequenced and assembled genetic material from these microcosms.

## Material and methods

### Sampling

A soil sample from the top 21 centimeters of the ground was collected in July 2010 from the Park Grass experiment (Vogel et al., 2009), Rothamsted Research, Hertfordshire, UK, using sterile manual corers (10 cm diameter) and was placed in sterile plastic bags, sealed and placed at room temperature 24 h while it was transported to Lyon, France. The sample was then sieved (2 mm) and directly used for the microcosm experiment.

### Microcosm conditions

Microcosms were done in triplicates (50 g of soil in each microcosm), stored at room temperature (except for the high temperature condition), without light, and were hermetically closed during the experiment. Following ESCs applied to each microcosm: ESC C (control): 5 ml of purified water was sprayed for control; ESC 1: 5 ml of purified water with ethanol (20% of the volume) was sprayed for ethanol enrichment; ESC 2: 5 ml of purified water with NaCl (30 g/L) was sprayed for salt enrichment #1; ESC 3: 5 ml of purified water with NaCl (300 g/L, salt saturation) was sprayed for salt enrichment #2; ESC 4: 5 ml of purified water was sprayed and then microcosms were incubated at 37°C for high temperature condition; ESC 5: 5 ml of purified water was sprayed and then, the microcosm atmosphere was replaced by nitrogen gas for nitrogen atmospheric condition; ESC 6: 5 ml of purified water with diesel (for a final concentration of 50 g/kg of soil) was sprayed for diesel enrichment; ESC 7: 5 ml of purified water with heavy metals (zinc, cadmium, nickel, and cobalt, for a final concentration of 0.2 g/kg of soil for each metal) was sprayed for heavy metals enrichment #1; ESC 8: 5 ml of purified water with heavy metals (zinc, cadmium, nickel, and cobalt, for a final concentration of 2 g/kg of soil for each metal) was sprayed for heavy metals enrichment #2; ESC 9: 5 ml of purified water with inorganic mercury (HgCl_2_ for a final concentration of 0.02 g/kg of soil) was sprayed for mercury enrichment #1; ESC 10: 5 ml of purified water with inorganic mercury (HgCl_2_ for a final concentration of 0.2 g/kg of soil for each metal) was sprayed for mercury enrichment #2. Note that microcosms were aerated (by opening the cap for about a minute) every week to renewing the normal air condition. For the nitrogen condition (ESC 5), this step was immediately followed by replacing the air with nitrogen gas.

### DNA extraction, quantification, and fingerprint analyzes

After 4 months of incubation, DNA samples were extracted from 0.5 g of soil using the MP BIO 1O1 fast prep (Biomedical, Eschwege, Germany), a protocol known to be relatively efficient for this particular soil (Delmont et al., 2011b). Samples were purified using GFX columns (GE Healthcare) (final volume of 40 microliters) and the DNA was finally quantified using the Qubit® (1.0) Fluorometer. A minimum of six DNA extractions were used to estimate the quantity of DNA extracted and purified from each microcosm. Ribosomal intergenic spacer analyzes (RISA) were done for each microcosm to study condition reproducibility in term of microbial community relative composition. See (Delmont et al., 2011b) for more details about RISA analyses. RISA profiles were reproducible for all ESCs but the mercury enrichment #2 were an outlier microcosm was detected (Figure S1).

### Metagenomic sequencing

A minimum of 10 μg of DNA were used to generate metagenomic libraries for each Roche/454 pyrosequencing run on a 454 pyrosequencer (GS FLX Titanium Series Reagents; Roche 454; Shirley, NY, USA). In a first phase, pair-end sequencing was done on duplicate microcosms of each ESC (about one million reads per microcosm). As an exception, the three replicates of the mercury enrichment #2 were sequenced due to a low reproducibility of one microcosm observed using RISA profiles. Thus, 23 metagenomic data were generated. In a second phase, a mate-pair sequencing effort with 3 kb of gap was done for duplicates corresponding to 3 promising ESCs: heavy metals enrichment #2 (one million reads each), mercury enrichment #1 (two million reads each) and the outlier replicate of the mercury enrichment #2 (one million reads). Duplicates corresponding to the ethanol enrichment were also further sequenced, however, due to the high fragmentation of the DNA recovered from this ESC, additional sequencing was done with paired-end library preparation instead of mate-pair.

### Data analyses

Raw metagenomic reads were annotated using MG-RAST (Meyer et al., 2008). Detected functions and taxa were normalized to 100% in each sample, and a Kruskal–Wallis test was performed for Pfams and genera using the R package vegan (Oksanen et al., 2007). Metagenomic data were also sequentially assembled (Newbler software v.05, assembly requirement of 96% identity over a minimum length of 100 nt, with reads limited to one contig/scaffold). Coverage, GC-content and tetranucleotide frequency information was used to bin contigs/scaffold into draft genomes. Visualization of this information was done using in-house Python programs. Genomes and other genetic structures reconstructed were subsequently annotated using RAST (Aziz et al., 2008) and visualized using Artemis and DNAPlotter (Carver et al., 2009). Completions estimates were done using a collection of single-copy genes (Wu and Eisen, 2008). Metagenomic datasets were then mapped to these genetic structures (using CLC v.6 and a mapping requirement of 97% and 90% identity over the full read length) to estimate their distribution in the different microcosms as well as in the natural microbial community (Delmont et al., 2012) and in communities from distant soil biomes (Fierer et al., 2012). Positive detection was defined when a minimum of 10 reads from the targeted structure were detected in the selected dataset. Gephi v0.8.2 (Bastian et al., 2009) was used to generate functional networks (Force Atlas 2) connecting different collections of reconstructed genomes and an exhaustive list of genomes affiliated to the same taxonomy available in NCBI in April 2014: 24 *Streptomyces*, 18 *Chloroflexi*, 10 *Acidobacteria*, 6 *Rhodanobacter*, and 3 *Sporolactobacillus*. In parallel, sequences related to the 16S rRNA gene were screened from metagenomic datasets using RDP (Cole et al., 2009) database through MG-RAST (Meyer et al., 2008) with a minimum of 200 nt of alignment and 90% identity. Sequences so collected were partitioned by genus and independently assembled on Newbler (100 nt alignment, 99% identity minimum, reads limited to one contig) to create consensus sequences. The metagenomic dataset is publically available at http://www.genomenviron.org/Projects/METASOIL.html. Draft genomes are publically available at http://dx.doi.org/10.6084/m9.figshare.1320632 and in the NCBI Bioproject PRJNA279807.

### 16S rRNA gene amplicons

A 16S rRNA gene amplification step was done for DNA pools extracted from the seven microcosms targeted during the second sequencing phase as well as one microcosm representing the control condition. Primers pA (5′ AGA GTT TGA TCC TGG CTC AG 3′) and pH (5′ AAG GAG GTG ATC CAG CCG CA 3′) were used for this amplification. PCR products were then gel-purified (GE Healthcare illustra GFX PCR DNA and Gel Band Purification Kit) and cloned in a TOPO-TA using the TOPO-TA cloning kit for Sequencing (Invitrogen). 96 clones for each microcosm condition were randomly selected and inoculated in a 96 well plate. After TOPO-TA vector extraction, inserts were sequenced with Sanger Technology (Beckmann Coulter Genomics). Both primer M13for (5′ TGT AAA ACG ACG GCC AGT 3) and primer M13rev (5′ CAG GAA ACA GCT ATG ACC 3′) reactions were done. Sequences obtained with forward and reverse reactions were assembled and vector-purified with Seqman Software (Lasergen Seqman DNASTAR). Consensus sequences were then compiled by dataset for further data analysis.

### Taxonomical inference

Genomic taxonomical affiliation was inferred using information extracted from (i) global sequence similarity with the RAST collection of genomes (Aziz et al., 2008), (ii) full or fragmented 16S rRNA genes present in assembled genomes, (iii) sequences related to 16S rRNA gene assembled after their screening at the genus level in unassembled metagenomic datasets, and (iv) 16S rRNA gene amplicon datasets.

## Results and discussion

### ESCs stimulate the reconstruction of genetic material from the soil microbiome

**Figure 1:**
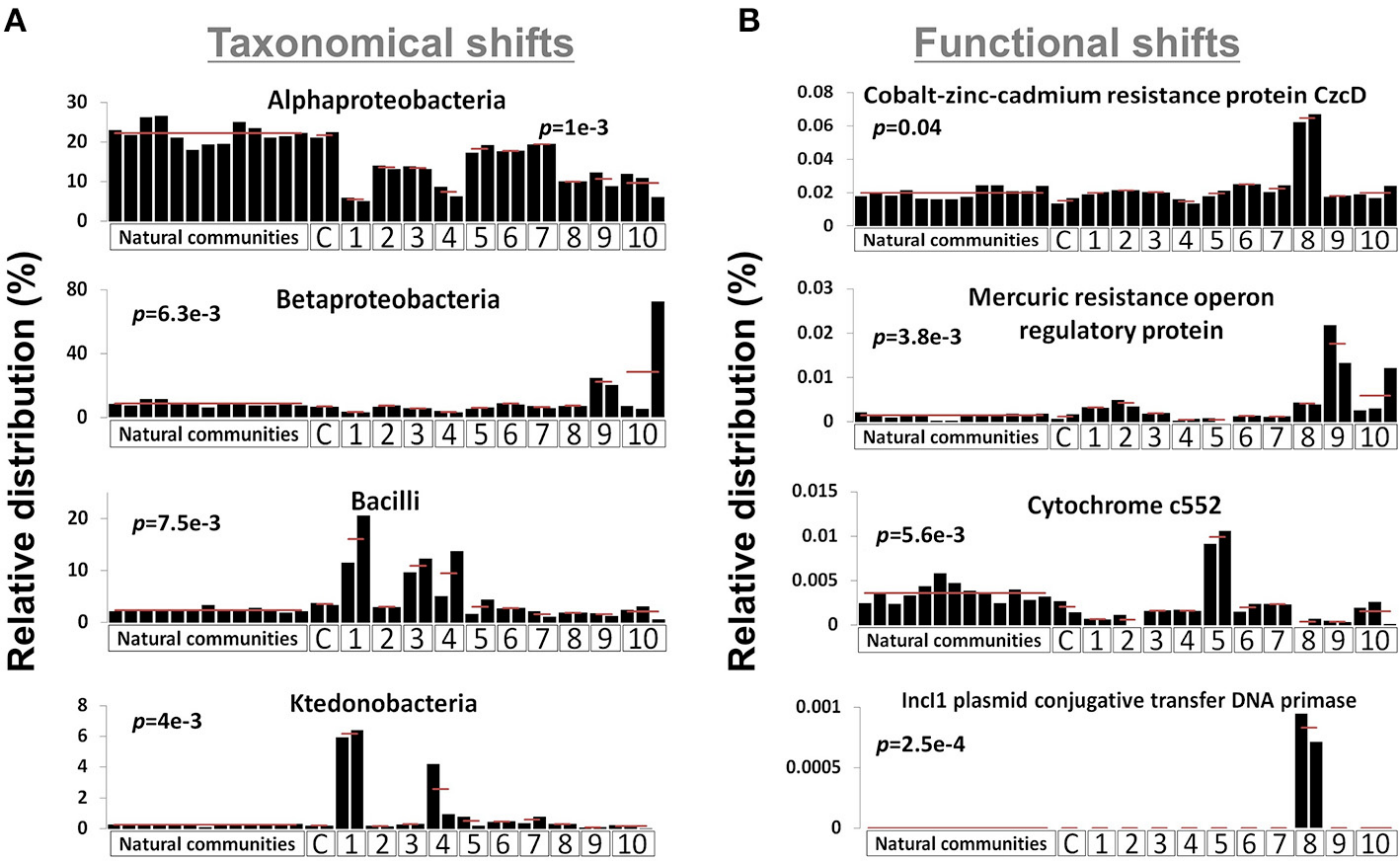
**Graphs represent the relative distribution of 4 bacterial taxa using M5NR databases (A) and Pfams (B) in the 36 metagenomic datasets when annotated in MG-RAST**. *E*-value cut-off was defined as 10-5. *P*-values were defined using distribution variations between conditions (Kruskal-Wallis test). X-axes identify different ESCs: C, control; 1, ethanol; 2, salt #1; 3, salt #2; 4, 37°C; 5, nitrogen; 6, diesel; 7, heavy metals #1; 8, heavy metals #2; 9, mercury #1; 10, mercury #2.

We sequenced the genetic material of two microcosms for each ESC as well as the outlier microcosm replicate of the mercury enrichment #2 (see Figure S1), thus generating 23 metagenomic datasets. 1.05 (± 0.17) million pyrosequencing reads of about 350 nucleotides were generated for each microcosm, representing a total of 24.05 million reads. We combined the 23 unassembled metagenomic dataset with the 13 dataset previously generated from the natural community of the same site for community composition comparison purposes (Delmont et al., 2012). The latter dataset was generated with the same sequencing strategy and provides background regarding the natural and methodological fluctuation of the Park Grass soil microbiome. MG-RAST identified 4,081 genera and 8,541 distinct protein families (Pfams) from the combined datasets (Tables S1, S2). Among these, several taxonomical groups and Pfams varied significantly between different conditions (Kruskal-Wallis test, *p*-value < 0.05), indicating that the microcosm strategy initiated a shift in the composition of the initial microbial community (Figure 1). Overall, the proportion of Alphaproteobacteria decreased in all ESCs, which benefited various ESC-specific taxa (e.g., *Ktedonobacter*, *Bacilli*, Betaproteobacteria). We detected positive selective pressure effect on functions directly related to ESCs: the Pfam related to Cobalt-zinc-cadmium resistance protein CzcD was more prevalent under the heavy metal enrichment #2, anaerobic cytochrome c552 was higher in soil under nitrogen gas atmosphere, and mercury resistance operon regulatory protein was higher in the mercury enrichments. Moreover, some functions were reproducibly detected only under specific conditions, such as IncI1 plasmid conjugative transfer DNA primase in microcosms from the heavy metal enrichment #2.

**Figure 2:**
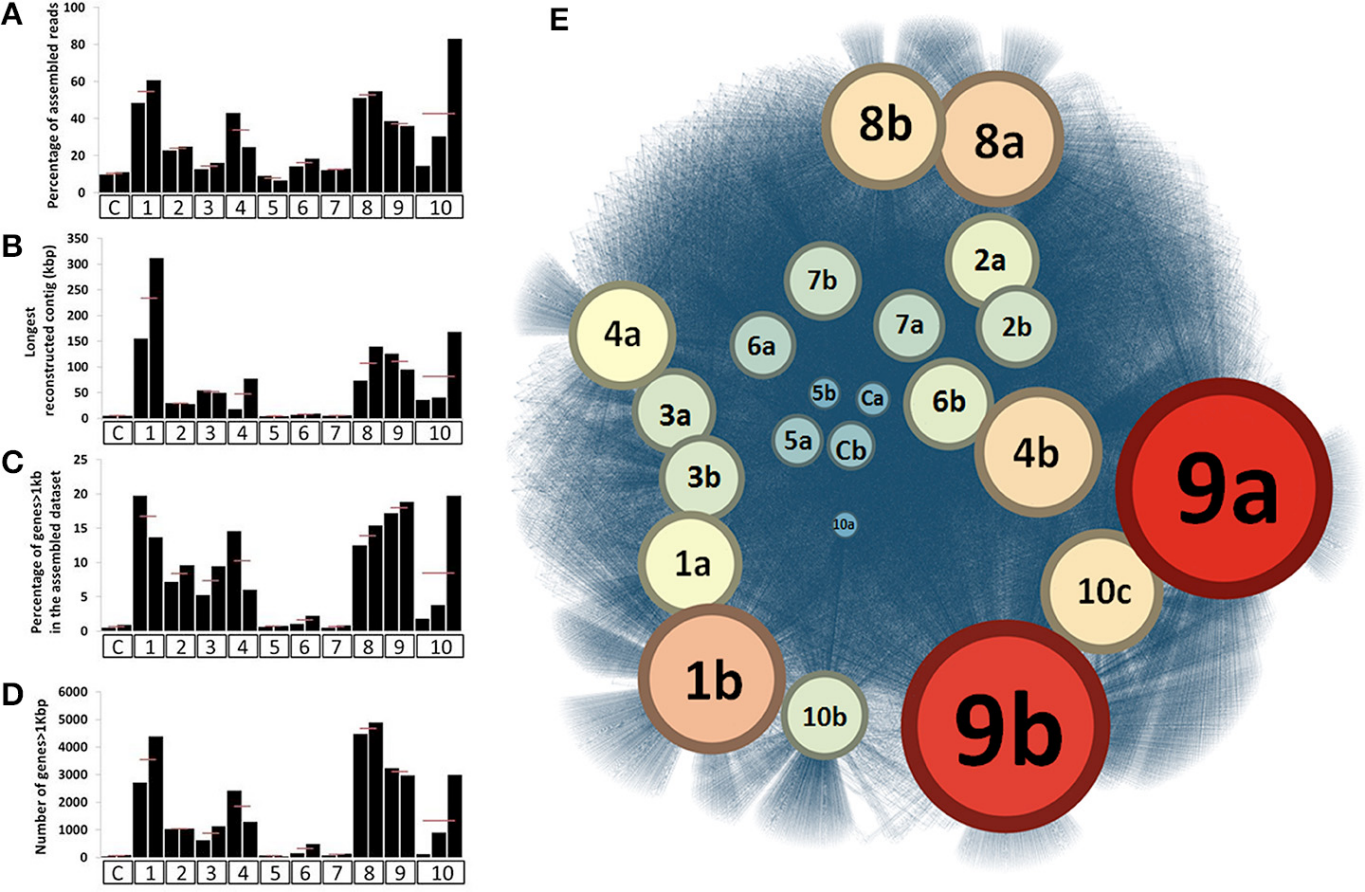
**Panels (A,B) represent the relative percentage of assembled reads and longest reconstructed contig after assembling the 23 datasets, respectively**. Panels **(C,D)** represent the number and percentage of genes longer than 1kb recovered from these assemblies, respectively. For **(A–D)**, X-axes identify different ESCs. Finally, **(E)** represents a functional network linking the 23 assembled metagenomic datasets and the 24,291 distinct functions annotated from these assemblies. The network was generated using Gephi and Force Atlas 2 and represents a total of 85,188 connections. Datasets are colored and have a size depending on the number of different functions they are connected to. In all panels, ESCs represent the following conditions; C, control; 1, ethanol; 2, salt #1; 3, salt #2; 4, 37°C; 5, nitrogen; 6, diesel; 7, heavy metals #1; 8, heavy metals #2; 9, mercury #1; 10, mercury #2.

For most ESCs, we observed a decrease in the total amount of DNA, suggesting overall population declines throughout the incubation period (Figure S2). DNA extraction yield varied from 14.6 (± 2.7) μg to 0.7 (± 0.4) μg per gram of soil depending on the ESC while it was about 25 μg for the untreated sample. However, the taxonomical and functional shifts indicated that the ESCs impacted different parts of the soil microbiome, providing access to distinct genomic populations through assembly. We assembled each metagenome obtained from microcosms separately, and RAST annotation identified a total of 386,684 genes (average length of 451 nt) in contigs ranging from 0.1 to 300 kb, with 59% of them longer than 1 kb. Assembly efficiency and average length of genes were substantially enhanced for many of the ESCs in comparison to our controls (Figures 2A–D). Interestingly, although some conditions, such as Nitrogen headspace, provided a different community structure, they did not enhance the assembly, suggesting that a large number of microbial populations were co-selected. 52.4% of the genes were successfully annotated in RAST (Table S3) and affiliated to 24,291 different functions, most of which were related to Bacteria. We detected three times more functions through ~400 thousand assembled genes with RAST, compared to ~36 million unassembled reads using MG-RAST. This functional recovery represents a significant achievement in comparison to what was previously detected from the direct sequencing effort of the Park Grass microbial community (Delmont et al., 2012).

Our network analysis connecting 24,291 functions to microcosms they originate demonstrated the efficacy of multiple ESCs to stimulate the recovery of different functional pools (Figure 2E). In particular, the mercury enrichment #1 provided a total of 3,155 unique functions from 68,450 assembled genes, representing an 18-fold increase compared to controls (176 unique functions only). Overall, this analysis showed that ethanol, heavy metals and mercury ESCs resulted in highest rates of gene and function recovery through metagenomic assemblies.

### We assembled 1% of the soil microbiome and recovered novel bacterial genomes

**Figure 3:**
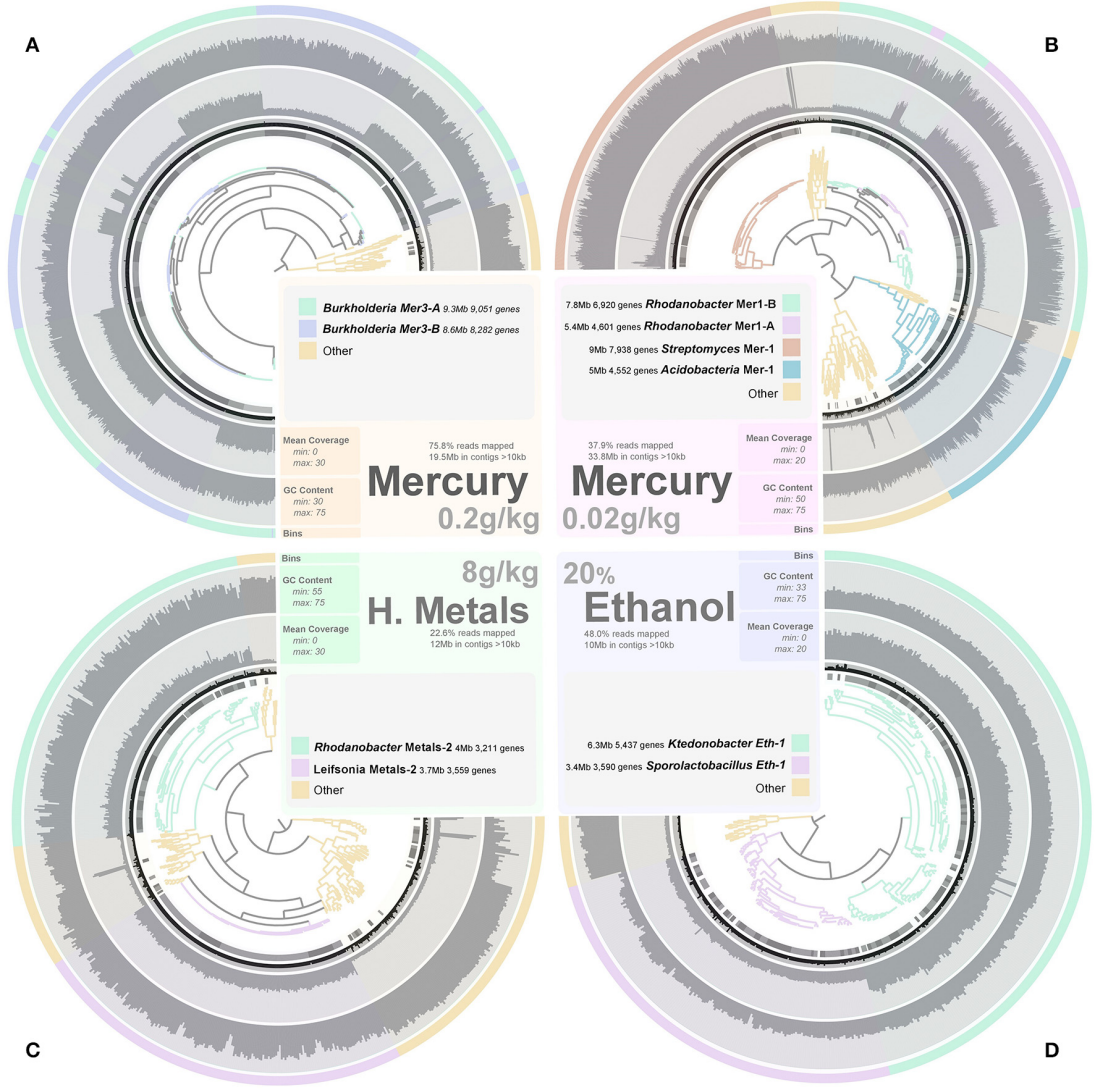
**Panels (A–D) exhibit the metagenomic assemblies (genetic structures reaching 10 kb only) recovered from the Mercury enrichments 2 (19.5 Mb) and 1 (33.8 Mb), heavy metals enrichment 2 (12 Mb) and ethanol enrichment (10 Mb), respectively**. We applied a mapping requirement of 97% identity to estimate coverage values. Genetic structures are organized in trees based on their tetranucleotide frequency (Euclidean distance) and were subsequently fragmented into sections of 20 kb displaying the same color in the first outer cycle. Therefore, each section in the tree represents a genetic structure ranging from 10 to 20 kb (length is displayed in the second outer cycle in black). Mean coverage (third outer cycle) and GC-content (forth outer cycle) are display for each section to assess the coherence of clusters. Finally, draft genomes determined from these assemblies are presented in the last outer cycle as well as in the tree itself.

We performed additional sequencing for seven microcosms that showed highest potential for recovery of genomes (ethanol enrichment, heavy metals enrichment #2 and mercury enrichments #1 and #2) based on metagenomic assembly scores (Figure 2). Assembly of the additional data resulted in genetic structures up to 4 Mb and lead to a total recovery of 334,576 coding genes and 3,820 RNAs from these seven microcosms using about 6 Gb of sequencing. Overall, we recovered 540 Mb of genetic material that harbors 559,555 genes (average length of 466 nt, includes 56,228 genes longer than 1,000 nt) representing 29,176 functions. This genetic material constitutes 1–2.5% of this soil microbiome at 97–90% identity cut-off, respectively. Ninety-seven percent identity cut-off complies with strain variation and sequencing errors and was selected for downstream analysis of the Park Grass soil microbiome. We used a tetranucleotide frequency-based supervised binning approach to identify draft genomes using contigs and scaffolds longer than 10 kb (Figure 3). In most cases, clusters displayed stable coverage and GC-content scores. Contigs that make up draft genomes of similar taxonomical affiliation most of the time organized in the same tetranucleotide frequency cluster, or showed consistent coverage when they were split into multiple clusters (Figure 3).

We determined a total of 17 draft genomes from these microcosms. Table 1 reports the functional properties and taxonomical affiliations of these genomes, which represent *Leifsonia* (*n* = 2), *Rhodanobacter* (*n* = 5), *Acidobacteria* (*n* = 2), *Sporolactobacillus* (*n* = 2), *Ktedonobacter* (*n* = 1), *Streptomyces* (*n* = 3), and *Burkholderia* (*n* = 2). They carry between 2,637 and 9,051 genes and possess a global GC-content ranging from 47.3% to 71%. Our analysis of single-copy gene families revealed that 11 of 17 bacterial genomes with no close relatives in their microcosms were 97% complete in average (Table 1). In contrast, the same analysis for the remaining 6 draft genomes that co-occur with their close relatives in microcosms they were found (i.e., two *Streptomyces* genomes and the two *Burkholderia* genomes) estimated that they were 61% complete in average, despite their large sizes (up to 9.3 Mb and 9 Mb for *Burkholderia* and *Streptomyces*, respectively). As an alternative approach we compared these draft genomes to the best matching reference genomes available using the best-hit function implemented in RAST. Our analysis indicated that the two *Streptomyces* draft genomes (Mer-2-A and Mer-2-B) covered 81% and 83% of the gold standard genome *Streptomyces avermitilis* MA-4680, respectively. Similarly, although single-copy gene analysis estimated only 45% and 36% completion for the two *Burkholderia* draft genomes, they covered 87–90% of the gold standard *Burkholderia xenovorans* LB400 chromosome 1. These contrasting findings suggest that draft genome completion estimates based on single-copy gene families can underestimate the rate of recovery when multiple closely related bacterial genomes are present in the assembly.

**Table 1:** **General information related to reconstructed genetic structures from metagenomic datasets related to seven microcosms**.

**Environmental stress conditions**	**Genetic structures recovered (and taxonomical affiliation)**	**Microcosm relative proportion (%)**	**Natural relative proportion (%)**	**Relative *in situ* enrichment**	**Number of contigs or scaffolds**	**Structure coverage in microcosm**	**GC content (%)**	**Genetic structure size (Mb)**	**Different tRNA synthetases**	**Completion estimation score**	**Number of detected genes**	**Percentage hypothetical proteins (%)**
Heavy metals 8 g/kg Microcosm 1	*Leifsonia* Metals-1	7.5	0.00012	6.2E+04	4 scaffolds	30X	67.8	3.8	20	0.97	3,681	27.6
	*Rhodanobacter* Metals-1	14.1	<0.0001	>1.4E+05	3 scaffolds	50X	68	4	19	1	3,600	27.2
	Plasmid	0.5	<0.0001	>5.2E+03	1 scaffold	100X	63.4	0.073	0	N/A	86	44.2
Heavy metals 8 g/kg Microcosm 2	*Leifsonia* Metals-2	8.9	0.00013	7.0E+04	5 scaffolds	30X	67.8	3.7	20	0.94	3,559	26.5
	*Rhodanobacter* Metals-2	9.4	0.0008	1.2E+04	30 scaffolds	40X	68	4	19	0.97	3,211	28.7
	Plasmid	0.5	<0.0001	>4.7E+3	1 scaffold	100X	63.5	0.072	0	N/A	82	42.7
Ethanol 20% Microcosm 1	*Sporolactobacillus* Eth-1	6.4	<0.0001	>6.4E+04	49 contigs	25X	47.3	3.4	20	0.97	3,590	31.9
	*Ktedonobacter* Eth-1	28.9	<0.0001	>2.9E+05	83 contigs	60X	50.4	6.3	19	1	5,437	43.4
Ethanol 20% Microcosm 2	*Sporolactobacillus* Eth-2	13.9	<0.0001	>1.4E+05	42 contigs	70X	47.3	3.3	20	1	3,508	32.3
Mercury 0.02 g/kg Microcosm 1	*Acidobacteria* Mer-1	4.0	<0.0001	>4E+04	19 scaffolds	15X	58.1	5.0	20	0.84	4,552	39.2
	*Rhodanobacter* Mer-l-A	12.6	0.00034	3.7E+04	14 scaffolds	60X	63.9	5.4	19	0.94	4,601	29.2
	*Rhodanobacter* Mer-l-B	5.0	0.00038	1.3E+04	24 scaffolds	20X	64.9	7.8	19	1	6,920	28.2
	*Streptomyces* Mer-l-A	8.9	0.00042	2.1E+04	11 scaffolds	25X	71	9.0	18	1	7,938	35
	Mer operon structure	0.6	<0.0001	>5.7E+03	1 scaffold	50X	70.5	0.309	0	N/A	313	63.9
	Bacteriophage	1.3	<0.0001	>1.3E+04	1 scaffold	670X	65.1	0.056	0	N/A	87	86.2
Mercury 0.02 g/kg Microcosm 2	*Acidobacteria* Mer-2	7.4	0.00096	7.7E+04	1 scaffold	25X	58	4.8	14	1	4,293	36.4
	*Rhodanobacter* Mer-2	11.5	0.0008	1.4E+04	6 scaffolds	60X	64.8	4.6	20	0.97	4,032	28.1
	*Streptomyces* Mer-2-A	8.2	0.00061	1.3E+04	46 scaffolds	25X	70.9	9.0	20	0.45	7,732	35.8
	*Streptomyces* Mer-2-B	3.8	0.011	3.5E+02	169 scaffolds	15X	70	7.8	20	0.48	5,882	34.6
	Mer operon structure	1.0	<0.0001	>1E+04	1 scaffold	80X	70.5	0.309	0	N/A	310	64.2
	Bacteriophage	7.7	<0.0001	>7.7E04	1 scaffold	1500X	64.4	0.156	0	N/A	235	90.2
	Bacteriophage	0.7	<0.0001	>7.3E+03	1 scaffold	180X	65.1	0.142	15	N/A	201	91.5
Mercury 0.2 g/kg Microcosm 3	*Burkholderia* Mer3-A	25.1	0.00024	9.5E+04	6 scaffolds	75X	61.1	4.2			4,018	21.2
	2 chromosmes and	24.9	0.00017	1.5E+05	4 scaffolds	75X	61.7	4	17	0.45	3,783	28.2
	1 mega-plasmid	7.7	0.000096	8.0E+04	2 scaffolds	75X	59.9	1.1			1,250	48
	*Burkholderia* Mer3-B	7.9	0.0079	1.0E+03	7 scaffolds	25X	62	4.3			4,070	20.2
	2 chromosmes and	6.4	0.0056	1.2E+03	6 scaffolds	25X	61.2	3.2	17	0.36	3,019	25.3
	1 mega-plasmid	2.3	0.0014	1.6E+03	3 scaffolds	25X	60.3	1.1			1,193	37.2

Taxonomical affiliation was estimated based on 16S rRNA gene sequence (when present) and global genomic similarity with a set of reference genomes through RAST. The Mer operon structure is carrying a set of genes related to mercury resistance.

The relative proportion of the reads associated with these genomes ranged between <0.0001% and >0.01% of the total reads sequenced from the original soil site [>12 million reads available (Delmont et al., 2012)] while they represented 2–58% of the reads recovered from microcosms after 4 months of treatment. Therefore, these genomes were enriched by several orders of magnitude during the incubation period, in contrast to the overall decline of total DNA observed after incubation. Genomes reconstructed from the ethanol enrichment condition were not detected in the natural community, with one of them related to the family *Ktedonobacteraceae*. Until now, this lineage had only one representative genome sequenced due to the difficulty cultivating this branch of the tree of life (Chang et al., 2011). This result emphasizes the interest of investigating rare members of the soil biosphere to recover the genomic content of novel taxonomical lineages. The recovery of this draft genome (6.3 Mb) would have been impossible with canonical approaches that focus solely upon post-sequencing data treatment.

In addition, we observed three phage infections from the two microcosm replicates of mercury enrichment #1, which were affiliated with the *Streptomyces* population based on the clustering tetranucleotide frequencies. Despite its small size of 156 kb, the most dominant phage recruited 7.68% of all reads in the metagenomic data generated from the second microcosm representative of this ESC and reached 1,500× coverage, which was 38 times more frequent than the two dominant *Streptomyces* genomes reconstructed from the same microcosm. Functions for most genes carried by these three phages were unknown.

Overall, we recovered from one to four bacterial genomes, along with thousands of orphan genetic structures in each targeted microcosm, validating the strategy reconstructing a soil metagenome one ESC at the time.

### Different mega-plasmids confer mercury resistance in coexisting Burkholderia populations

**Figure 4:**
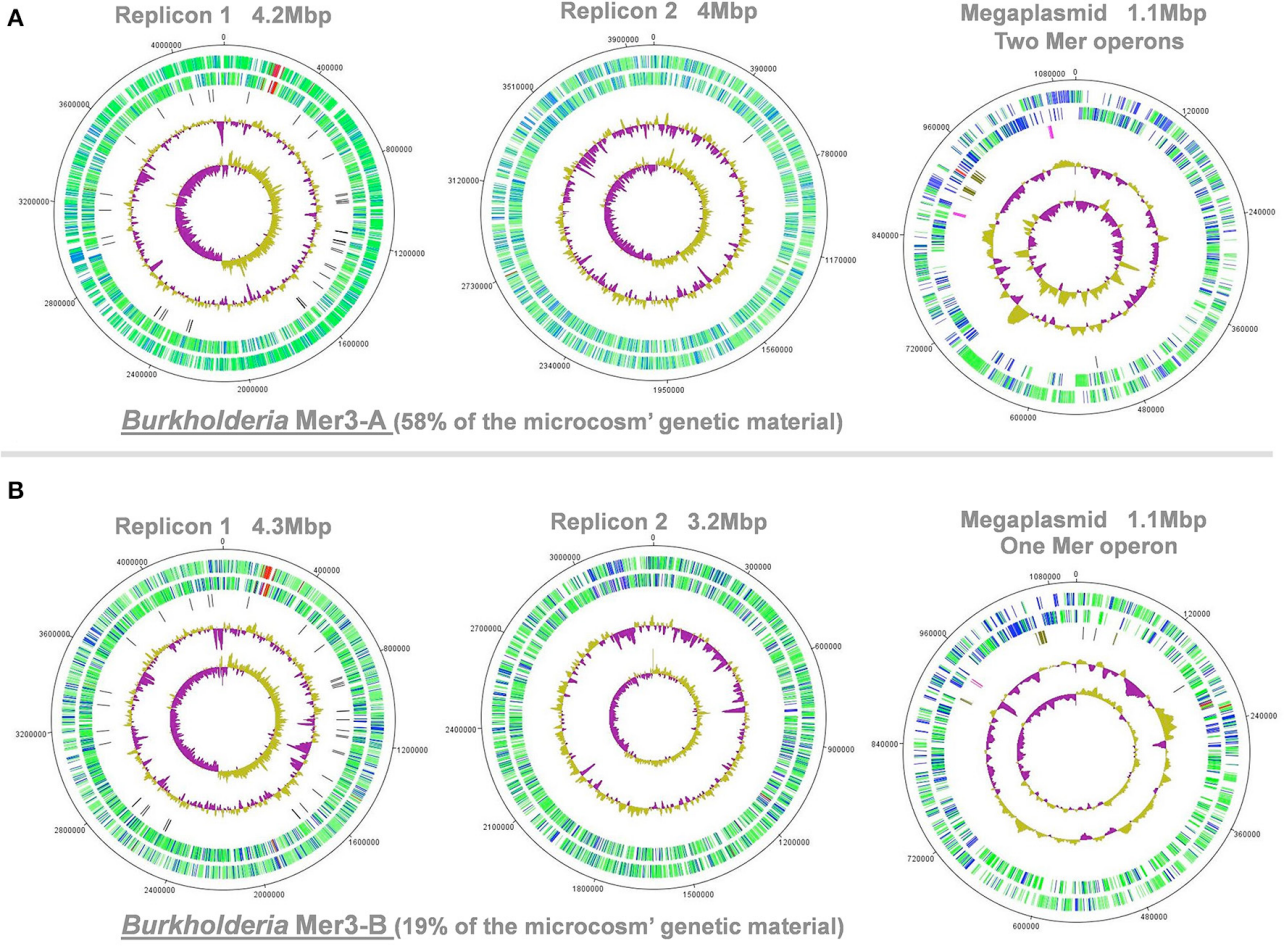
**Example of the two Burkholderia genomes reconstructed from the third replicate of mercury enrichment #2**. Panels **(A,B)** represent replicons and mega-plasmid from Burkholderia Mer3-A and Mer3-B, respectively. Artemis and DNAPlotter were used to visualize the two replicons and mega-plasmid present in this microorganism. First (i.e., interior) and second circles represents GC skew and GC-content variations, respectively. Third circle represents the location of tRNAs (dark), conjugative (chestnut), and mercury (pink) related genes. Fourth and fifth circles represent genes of known (green) and unknown (blue) functions as well as genes related to flagellum (red) in the two possible frames.

Burkholderia populations often harbor a multi-replicon genome in nature (Chain et al., 2006). Here, we ordered and oriented scaffolds from the two *Burkholderia* genomes (mercury enrichment #2) using G+C skew and recovered two replicons and a megaplasmid for each of them (Figure 4). The observation of a well-defined G+C skew provided an additional support for the reliability of our genomic reconstructions. A similar genomic organization was observed between the two *Burkholderia* genomes (e.g., tRNAs and flagellar genes operon in the replicon 1) and no differences could be detected between their full-length 16S rRNA genes. On the other hand, most genes and functions were not shared between the two mega-plasmids (Figure S3). These mobile genetic elements also possessed a higher proportion of hypothetical proteins. Yet, mega-plasmids carried similar mercury resistance gene operons, suggesting a preponderant role in the ability of these coexisting *Burkholderia* populations to grow under the mercury ESC. Two mercury resistance operons found in *Burkholderia* Mer3-A (representing the most abundant population) further supports this suggestion. Similar operons have been found in plasmids worldwide (Barrineau et al., 1983; Osborn et al., 1997) while, to the best of our knowledge, not specifically in organisms affiliated to *Burkholderia.* However, these two populations may have acquired mercury resistance operons during the incubation period, and may not necessarily possess mercury resistance in the pristine soil.

### ESCs are not redundant and therefore can lead to a large genomic catalogue from soil

**Figure 5:**
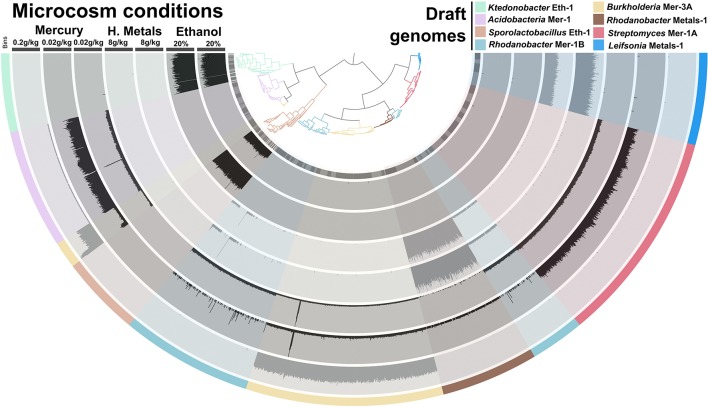
**Coverage of eight draft genomes (organized in a tree based on their tetranucleotide frequency (Euclidean distance) and fragmented into sections of 20 kb) in metagenomic data representing seven microcosms and four incubation conditions**. The data was generated during the first sequencing effort (paired-end sequencing). Maximum coverage varied between 30× and 50× depending on the data. Draft genomes are displayed in the outer cycle and the tree itself. Note that coverage discrepancies observed (e.g., for Burlholderia Mer-3A in the mercury enrichment #1) do not necessarily reflect a binning problem, as metagenomic reads that would have mapped to other genetic structures have a restrained target choice of 8 draft genomes in this analysis.

Different taxonomic groups in our soil sample adapted to different ESCs. For instance, while ethanol stimulated the growth of Firmicutes, heavy metals stimulated Proteobacteria and Actinobacteria. We generated a tetranucleotide frequency-based tree to display the distribution of 8 taxonomical groups that were present in our microcosms (Figure 5). Recovered genomes for each ESC displayed near-identical distribution patterns across replicate microcosms, demonstrating high reproducibility of ESC treatments. In most cases genomes that were enriched under a particular ESC, were not detected in other ESCs. An exception to this was *Rhodanobacter* Metals-1, which was abundant in heavy metals enrichment #2, and it was also detected in the mercury enrichment #1 (another heavy metal) in lower abundance. Overall, each ESC provided a unique set of genomes, suggesting that more genomes could be recovered by applying additional conditions to this soil. The recovery of distinct organisms by only changing the concentration of mercury also suggests that a fine gradient of concentrations may stimulate the recovery of more genomes from organisms that prefer a specific range. It is indeed difficult to estimate what fraction of the soil microbial community that can be targeted from such enrichment approaches, yet ESCs may be useful to build a larger catalog of genes and their genomic contexts found in soil.

### Recovered genomes harbor different functional pools driven by taxonomy

**Figure 6:**
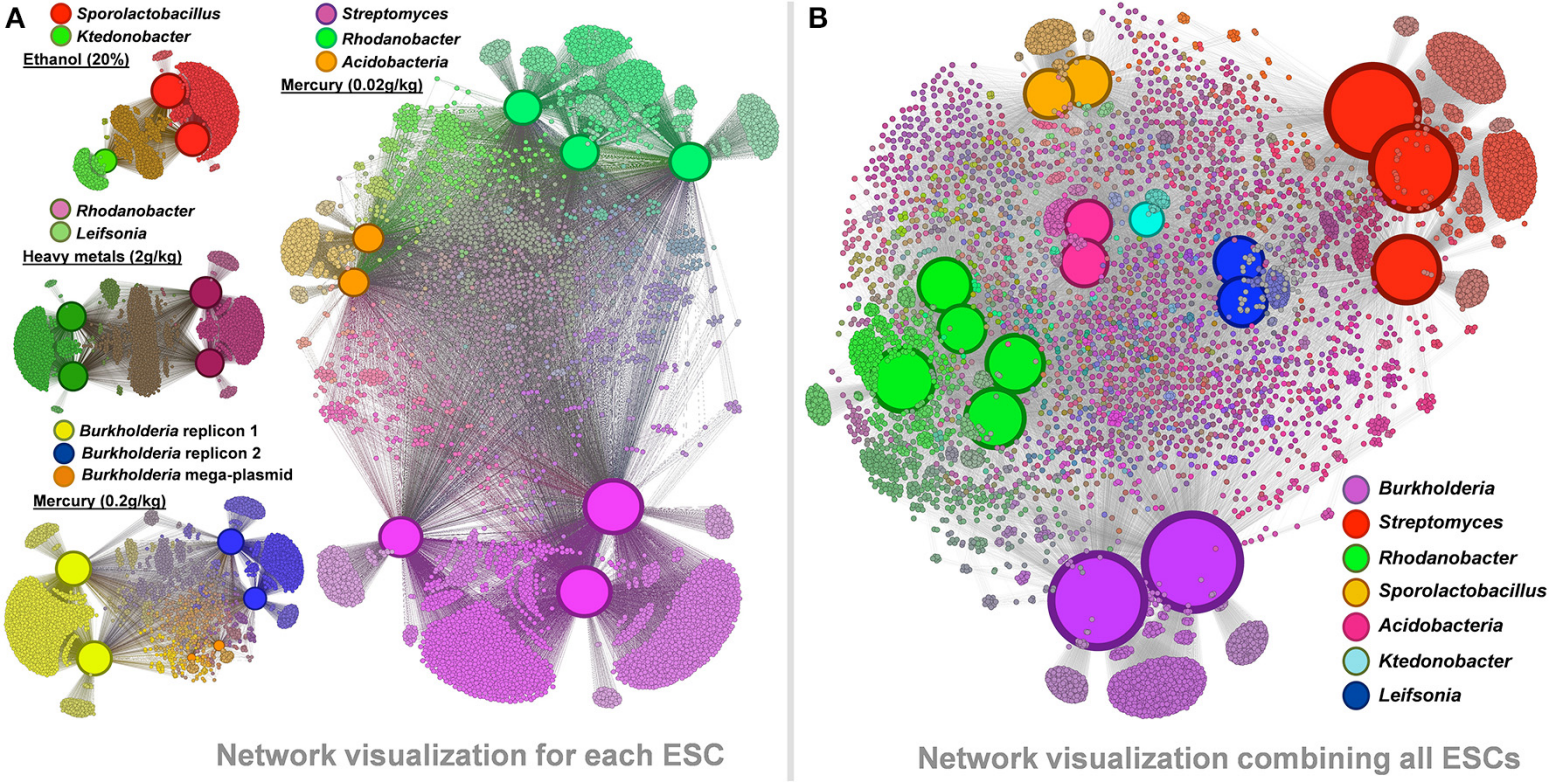
**Panel (A) functional networks linking genomes and their associated functions recovered from ethanol, heavy metals and mercury ESCs**. Panel **(B)** functional network linking the 17 recovered genomes and their associated functions (a total of 11,299 different functions were detected). Networks were generated using Gephi and Force Atlas 2. Node sizes are positively correlated to the number of connections in each network, leading to enhanced sizes for genomes. Note that genome sizes cannot be directly compared between networks. Genomic and functional nodes are colored by taxonomy and their genomic connections, respectively.

We performed a network visualization using observed functions in recovered genomes from each ESC (Figure 6A). Similar genomes recovered from multiple microcosms lead to the identification of large number of core functions (e.g., about 3,500 identical genes for the two recovered *Sporolactobacillus*), as well as smaller, microcosm-specific ones (mostly hypothetical proteins and mobile elements). Micro-diversity traits are commonly found in cultivated strains of similar taxonomy, including in those displaying identical 16S rRNA genes (Jaspers and Overmann, 2004; Pena et al., 2010). Note that only the consensus assemblies of each genome were compared between replicates but that the genomic populations recovered from our study are likely to be polyclonal, contrasting with most micro-diversity analyses performed using isolate collections. We also generated an overall functional network using all 17 genomes and 11,299 functions they entailed (Figure 6B). The network analysis highlights the functional similarity of the five *Rhodanobacter* genomes in spite of different ESCs they were found (combination of heavy metals vs. mercury alone). Each taxonomical group possessed unique functions (e.g., 2,088 and 2,545 functions only detected in *Burkholderia* and *Streptomyces* genomes, respectively).

### Genomic functional differences suggest distinct ecological roles within the soil microbiome

Our genomic collection contained seven genera (Gram+/Gram− ratio was close to 1) with distinct functions. A majority of these organisms possessed gliding and flagellar motility capacities. Moreover, they harbored different metabolic capacities (e.g., Entner-Doudoroff pathway, betaglucoside utilization, fructose utilization) and strategies to target iron (e.g., ton and tol transport system, siderophore assembly) (Table 2). The *Sporolactobacillus* genomes possessed more than 40 genes related to spore formation, none of these genes being detected in the other genomes. Derived from the same condition and representing an unusual functional trait for Bacteria, the *Ktedonobacter* genome has the potential to produce both vitamin K1 (Phylloquinone) and K2 (Menaquinone) from Chorismate. The three *Streptomyces* genomes carried genes related to ethylmalonyl-CoA pathway of C2 assimilation, spore pigment biosynthetic cluster, sigmaB stress response regulation, sialic acid metabolism and siderophore assembly. As a potential industrial interest, several novel polyketide synthase modules were detected in these genomes. The two *Burkholderia* species possessed genes for degrading benzoate, but only one was carrying genes for a chloroaromatic degradation pathway, while the other harbored genes for degrading chlorobenzoate. When focusing on the nitrogen cycle, all these populations had the functional potential to assimilate ammonia and most of them could perform nitrite and nitrate ammonification. On the other hand, only the *Sporolactobacillus* genomes possessed adequate genetic information to fix nitrogen from the atmosphere (operon nifDKHENBQU). This is the first time this taxon is linked to nitrogen fixation, which is an important ecological trait in soil systems (Peoples et al., 1995; Bothe et al., 2006; Prosser et al., 2006). Finally, one *Rhodanobacter* genome possessed the genes required to perform denitrification. *Rhodanobacter* is involved in the soil nitrogen cycle through denitrification (Green et al., 2012; Kostka et al., 2012) and our metagenomic-derived genomes also harbored this functional trait.

**Table 2:** **Some information (e.g., nitrogen cycle) related to the ecological role of the 17 reconstructed genomes in the natural microbial community**.

**Genomic structures**	**Type**	**Estimated genomic population in one gram of soil**	**Gliding bacterial motility**	**Flagellar motility**	**Entner-Doudoroff pathway**	**Beta-Glucoside metabo-lism**	**Fructose utilization**	**Exopoly-saccharide biosynthesis**	**Ton and Tol transport systems**	**Siderophore assembly**	**Denitrifi-cation**	**Nitrogen fixation**	**Ammonia assimilation**	**Nitrate and nitrite ammonifi-cation**	**Inorganic sulfur assimilation**
**HEAVY METALS 8 g/kg**															
*Leifsonia* Metals-1	Gram +	1,200	Yes	No	Yes	No	No	No	No	No	No	No	Yes	Yes	No
*Rhodonobacter* Metals-1	Gram −	<1,000	Yes	Yes	Yes	No	No	No	Yes	No	No	No	Yes	Yes	Yes
*Leifsonia* Metals-2	Gram +	1,300	Yes	No	Yes	No	No	No	No	No	No	No	Yes	Yes	No
*Rhodanobacter* Metals-2	Gram −	800	Yes	No	Yes	No	No	No	Yes	No	No	No	Yes	Yes	No
**ETHANOL 20%**															
*Ktedonobacter* Eth-1	Gram +	<1,000	No	No	No	No	Yes	No	No	No	No	No	Yes	No	No
*Sporolactobacillus* Eth-1	Gram +	<1,000	Yes	Yes	No	Yes	Yes	Yes	No	No	No	Yes	Yes	No	Yes
*Sporolactobacillus* Eth-2	Gram +	<1,000	Yes	Yes	No	Yes	Yes	Yes	No	No	No	Yes	Yes	No	Yes
**MERCURY 0.02 g/kg**															
*Acidobacteria* Mer-1	Gram −	<1,000	No	Yes	Yes	No	No	No	Yes	No	No	No	Yes	Yes	No
*Rhodanobacter* Mer-1.A	Gram −	3,400	Yes	Yes	Yes	No	No	No	Yes	No	Yes	No	Yes	Yes	No
*Rhodanobacter* Mer-1.B	Gram −	3,800	Yes	Yes	Yes	No	No	No	Yes	No	No	No	Yes	No	Yes
*Streptomyces* Mer-1	Gram +	4,200	No	No	Yes	No	No	No	No	Yes	No	No	Yes	Yes	Yes
*Acidobacteria* Mer-2	Gram −	960	No	Yes	Yes	No	No	No	Yes	No	No	No	Yes	Yes	No
*Rhodanobacter* Mer-2	Gram −	8,000	Yes	Yes	Yes	No	No	No	Yes	No	Yes	No	Yes	Yes	Yes
*Streptomyces* Mer-2.A	Gram +	6,100	No	No	Yes	No	No	No	No	Yes	No	No	Yes	Yes	No
*Streptomyces* Mer-2.B	Gram +	110,000	No	No	No	No	No	No	No	Yes	No	No	Yes	Yes	Yes
**MERCURY 0.2 g/kg**															
*Burkholderia* Mer3-A replicon 1	Gram −	2,600	No	Yes	Yes	No	No	No	Yes	No	No	No	Yes	Yes	Yes
*Burkholderia* Mer3-A replicon 2	Gram −	1,700	No	No	No	No	No	No	Yes	No	No	No	No	Yes	No
*Burkholderia* Mer3-A megaplasmid	Gram −	960	No	No	No	No	No	No	No	No	No	No	No	No	No
*Burkholderia* Mer3-B replicon 1	Gram −	79,000	No	Yes	Yes	No	No	No	Yes	No	No	No	Yes	Yes	Yes
*Burkholderia* Mer3-B replicon 2	Gram −	56,000	No	No	No	No	No	No	Yes	No	No	No	No	No	No
*Burkholderia* Mer3-B megaplasmid	Gram −	14,000	No	No	No	No	No	No	No	No	No	No	Yes	No	No

Moreover, population estimates are based on local blast on datasets related to the natural microbial community and the postulate of 109 cells per gram of soil.

### Enriched populations harbor functional traits undetected in culture representatives

**Figure 7:**
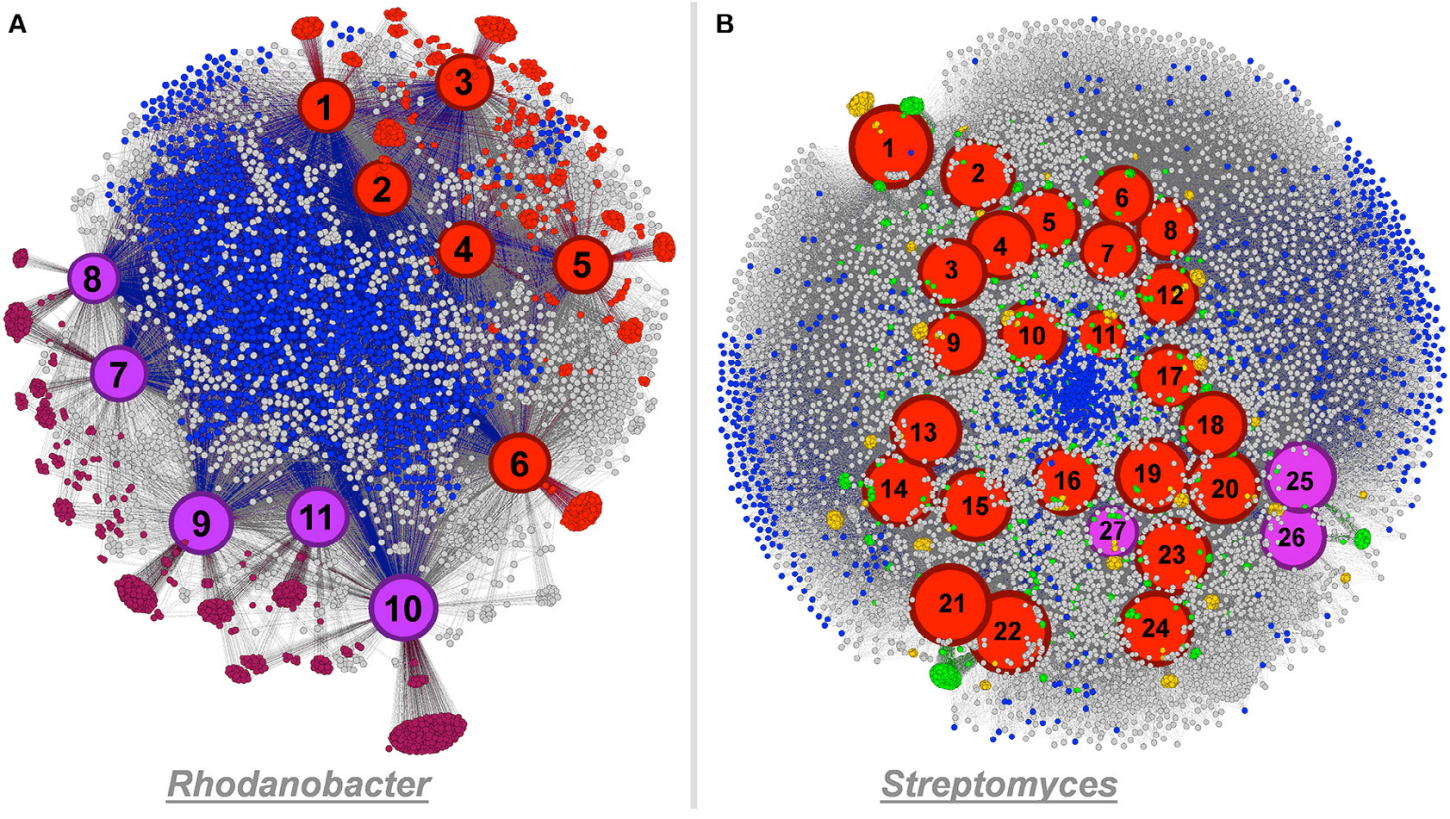
**Panel (A) functional network linking 11 Rhodanobacter genomes and their functionality (a total of 4,824 functions) using Gephi**. Culture-derived genomes are represented by red nodes: 1, *R. spathiphylli* B39; 2, *R. fulvus* Jip2; 3, R. sp. 115; 4, *R. denitrificans* 116- 2; 5, *R. thiooxydans* LCS2; 6, R. sp. 2APBS1. Metagenomic-derived genomes are represented by purple nodes: 7, R. Metals-1; 8, R. Metals-2; 9, R. Mer-1A; 10, R. Mer-1B; 11, R. Mer-2. Genomic node sizes are positively correlated to the number of connected functions. For the functional nodes, red and purple nodes represent functions detected only in one or more culture-derived or metagenomic-derived genome, respectively. Finally, blue functional nodes are detected in the 11 genomes. Panel **(B)** functional network linking 27 Streptomyces genomes and their functionality (a total of 11,032 functions) using Gephi. Culture-derived genomes are represented by red nodes: 1, *S. griseus* NBRC 13350; 2, *S. fulvissimus* DSM 40593; 3, S. sp. Sirex AA- E; 4, S. sp. PAM C26508; 5, *S. flavogriseus* ATCC 33331; 6, S. sp. GBA 94- 10; 7, S. sp. PVA 94- 07; 8, *S. albus* J1074; 9, S. sp. Tu6071; 10, *S. venezuelae* ATCC 10712; 11, *S. cattleya* DSM 46488; 12, *S. clavuligerus* ATCC 27064; 13, *S. violaceusniger* Tu 4113: 14, *S. rapamycinicus* NRRL 5491; 15, *S. bingchenggensis* BCW- 1; 16, *S. ghanaensis* ATCC 14672; 17, *S. albulus* CCRC 11814; 18, *S. collinus* Tu 365; 19, *S. davawensis* JCM 4913; 20, *S. hygroscopicus* 5008; 21, *S. lividans* 1326; 22, *S. coelicolor* A3(2); 23, *S. scabiei* 87.22; 24, *S. avermitilis* MA- 4680. Metagenomic-derived genomes are represented by purple nodes: 25, S. Mer- 1A; 26, S. Mer- 2A; 27, S. Mer- 2B. Genomic node sizes are positively correlated to the number of connected functions. For the functional nodes, blues nodes are detected in all genomes and yellow and green nodes are detected in one and two genomes, respectively.

We compared the genomes we recovered from our microcosms to genomes of cultivated organisms to identify novel functions. The five *Rhodanobacter* genomes recovered from our ESCs contained more than a thousand additional functions compared to the six culture-derived *Rhodanobacter* genomes currently available. These functions include secretion pathway proteins, membrane and outer membrane proteins, peptidases, a penicillin amidase, various transcriptional regulators, virulence proteins, chitin utilization and lignin degradation proteins as well as proteins related to cyanophycin metabolism (both cyanophycin synthase and cyanophycinase) and inositol catabolism. On the other hand, 997 functions were detected in all *Rhodanobacter* genomes and 1159 only in the culture-derived genomes. The network analysis displayed separate groupings of cultivated and metagenomic analysis-based *Rhodanobacter* genomes, possibly due to differences in methodologies (Figure 7A). As another example, the three newly recovered *Streptomyces* genomes provided 330 functions undetected in the 24 culture-derived *Streptomyces* genomes, which represent 3% increase of the 10,702 functions cataloged for this genus (Figure 7B). Novel functions included ABC transporters, Daunorubicin (a chemotherapy agent) resistance, a ferredoxin, four glycosyl hydrolase families, phenazine (an antibiotic) and pyochelin (a siderophore) biosynthesis proteins as well as pullulanase and neopullulanase (starch catabolism) genes. *Streptomyces* is a filamentous group of bacteria responsible for producing natural antibiotics that can be used in human and veterinary medicine (Malpartida and Hopwood, 1984). Antibiotic resistance in disease-causing bacteria is a growing concern (Thomson, 1999; Martinez and Baquero, 2000; Andersson and Hughes, 2010), and soil is a promising environment for new targets (Lin et al., 2015). Our recovery of *Streptomyces* draft genomes from multiple enrichment conditions suggests that enrichment studies could accelerate the discovery of novel bio-active compounds.

### Representation of recovered genetic structures in geographically distinct soil biomes

**Figure 8:**
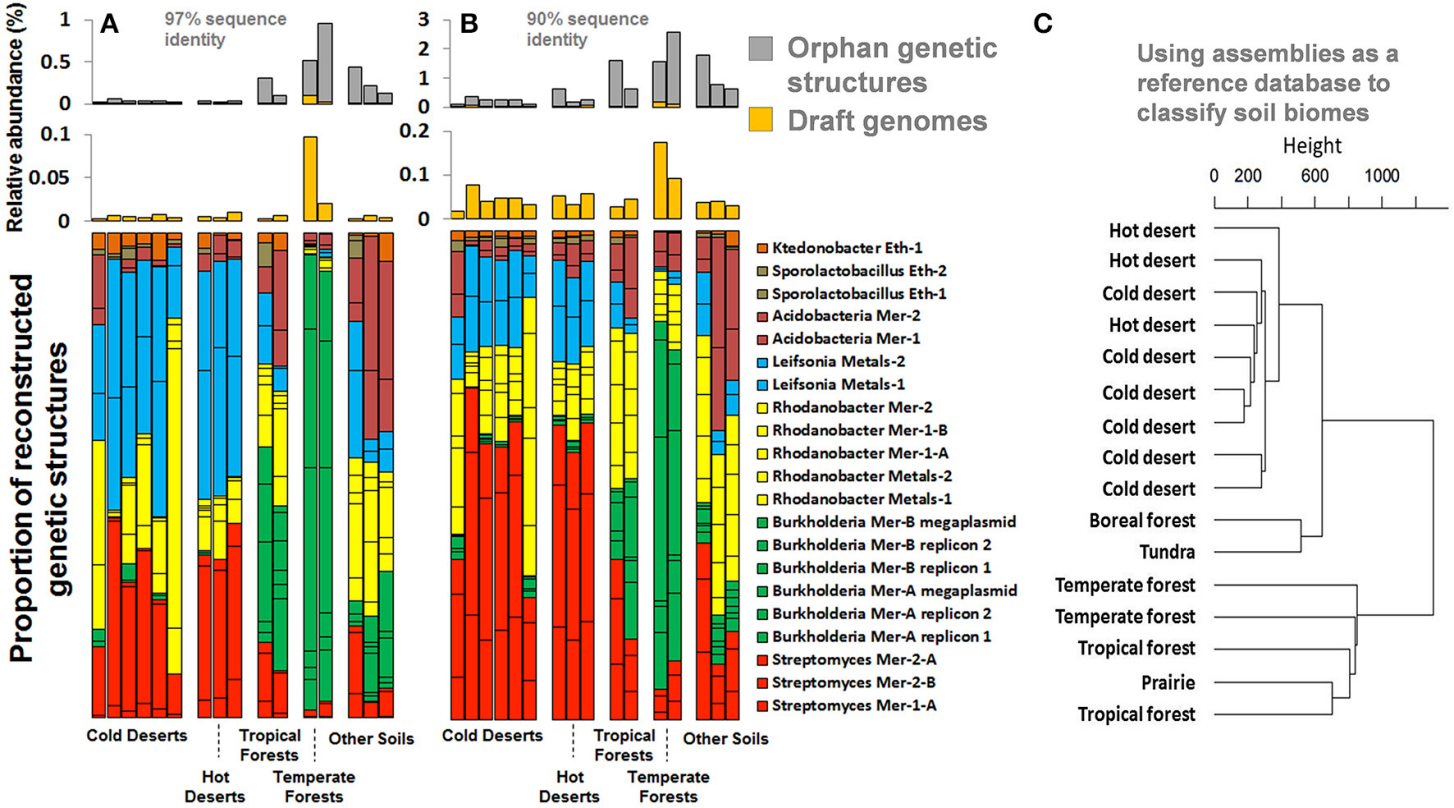
**Panels **(A,B)** represent the relative abundance and proportion of genomes and orphan genetic structures recovered from our ESCs in various soil biomes generated from Fierer et al. (2012), using a 97% and 90% sequence identity cut-off**. Genomes are colored based on their taxonomical affiliation at the genus level. Panel **(C)** represents the classification of the same samples using our assemblies (genomes and orphan genetic structures) as a reference database and a 90% sequence identity cut-off for mapping. The dendrogram was generated using Ward's method with Euclidean distances.

The 17 draft genomes we recovered from our ESCs represented about 0.03% of the natural Park Grass soil, based on metagenomic reads mapping at 97% identity cut-off. It is estimated that each gram of surface soil contains approximately 10^9^ bacterial cells, which suggests that the number of cells our genomes represent is up to 0.3 million per gram of this soil. We also traced the relative abundance of genomes we recovered in a metagenomic dataset of samples collected from distinct soil environments [cold and hot deserts, tropical and temperate forests, prairie, boreal forest, and tundra (Fierer et al., 2012)]. Although no genomes were recovered from these samples, Fierer et al. (2012) have reported different functional potentials based on short reads. Our recovered genomes recruited up to 0.1% and 0.2% of short reads from these samples at identity cut-offs of 97% and 90% (Figures 8A,B), suggesting that the taxonomic groups they represent are likely to occur globally. Moreover, genomes and orphan genetic structures (i.e., contigs that were not binned into genomes) recovered from the Park Grass soil occurred in different proportion between biomes (e.g., *Burkholderia* more prevalent in temperate forests), providing clear geographic patterns for the recruited soil microbiome fraction. We subsequently used these genetic structures (more than half a million contigs averaging 1 kb in length) as a first soil reference database of its kind to classify soil biomes and defined tree groups depicting deserts, high latitude soils and temperate soils (Figure 8C). These observations only partly agree with results obtained using reference databases lacking soil reference genomes that required low stringency annotations (Fierer et al., 2012) and provide a first case study on how soil reference genomic databases can assist analyzing and partitioning samples collected from this environment.

## Conclusion

Here we assembled 540 Mb of genetic material (including 17 draft genomes) from a soil sample using enrichment strategies. The assembled genetic material constituted about 1% of the original soil microbiome, and we achieved this using about 10 Gb of sequencing and standard bioinformatics approaches. We detected these draft genomes also in soil samples collected from distant locations. Although the recovered genetic structures from Park Grass explain only 1% of the diversity, considering the absence of any genomes recovered from soil through metagenomics to date, our study demonstrates the efficacy of pre-sequencing enrichment applications as a way to break into the vastly unexplored soil microbiome.

### Conflict of interest statement

The authors declare that the research was conducted in the absence of any commercial or financial relationships that could be construed as a potential conflict of interest.
