# Supplementary material for: Reconstructing rare soil microbial genomes using in situ enrichments and metagenomics
Source: Front Microbiol. 2015 Apr 30;6:358. doi: 10.3389/fmicb.2015.00358 (PMC4415585; doi:10.3389/fmicb.2015.00358)
Supplement: Supplementary file 3 [file TableS3.PDF]

	Controls		Ethanol enrich. 1		Nitrogen condition		Heavy metals enrich. 2		Mercury enrich. 1		Mercury enrich. 2			Total all
**General subsystem**	**Micro. 1**	**Micro. 2**	**Micro. 1**	**Micro. 2**	**Micro. 1**	**Micro. 2**	**Micro. 1**	**Micro. 2**	**Micro. 1**	**Micro. 2**	**Micro. 1**	**Micro. 2**	**Micro. 3**	**conditions**
**Amino Acids and Derivatives**	6	8	345	376	7	8	390	376	497	505	6	146	370	**5288**
**Carbohydrates**	0	4	408	492	0	2	441	403	548	545	0	143	385	**5488**
**Cell Division and Cell Cycle**	0	1	20	22	0	0	31	32	24	23	0	15	17	**372**
**Cell Wall and Capsule**	2	2	79	112	2	2	131	131	194	186	0	11	122	**1602**
**Cofactors, Vitamins, Prosthetic Groups, Pigments**	5	9	163	211	2	0	273	243	335	329	0	47	236	**3206**
**DNA Metabolism**	4	9	140	152	7	5	135	136	162	161	0	35	118	**1926**
**Dormancy and Sporulation**	0	0	24	70	0	0	3	3	13	11	0	1	2	**272**
**Fatty Acids, Lipids, and Isoprenoids**	5	1	88	105	2	0	82	76	106	84	1	13	80	**1235**
**Iron acquisition and metabolism**	0	0	2	2	0	0	5	5	53	40	2	0	31	**163**
**Membrane Transport**	3	3	65	67	4	2	122	117	150	115	0	5	110	**1127**
**Metabolism of Aromatic Compounds**	1	0	26	33	0	1	62	34	103	87	2	19	103	**648**
**Miscellaneous**	3	7	178	227	12	4	278	264	369	322	0	52	231	**3111**
**Motility and Chemotaxis**	0	0	39	67	0	0	74	71	83	87	0	0	71	**632**
**Nitrogen Metabolism**	0	0	13	19	0	1	27	27	47	39	0	3	33	**318**
**Nucleosides and Nucleotides**	0	5	104	119	6	2	102	106	137	128	2	59	96	**1500**
**Phages, Prophages, Transposable elements, Plasmids**	0	0	11	12	0	0	2	2	19	17	1	6	26	**122**
**Phosphorus Metabolism**	3	1	33	33	2	1	32	30	47	49	0	11	55	**502**
**Potassium metabolism**	1	3	9	9	3	0	15	13	16	17	0	0	15	**171**
**Protein Metabolism**	9	19	190	210	9	12	235	231	294	287	0	77	184	**3194**
**Regulation and Cell signaling**	2	3	33	32	4	2	54	57	87	86	0	6	74	**643**
**Respiration**	4	7	109	114	11	5	125	118	151	152	0	42	124	**1680**
**RNA Metabolism**	3	6	140	158	9	2	167	166	184	183	0	29	128	**2121**
**Secondary Metabolism**	0	0	13	14	0	0	7	8	13	10	0	5	0	**124**
**Stress Response**	13	7	80	88	12	9	135	129	176	152	5	46	109	**1590**
**Sulfur Metabolism**	0	2	22	46	3	2	44	40	57	59	0	8	47	**540**
**Virulence, Disease and Defense**	2	2	58	58	7	0	60	62	93	92	0	20	69	**867**
**Total number of annotated genes**	**66**	**99**	**2392**	**2848**	**102**	**60**	**3032**	**2880**	**3958**	**3766**	**19**	**799**	**2836**	**38442**

Table S3: Number of reconstructed genes assigned to a general RAST subsystem for the fraction of assembled metagenomic data sets related to the different microcosms and conditions
