# Supplementary figures and images for: Reconstructing rare soil microbial genomes using in situ enrichments and metagenomics
Source: Front Microbiol. 2015 Apr 30;6:358. doi: 10.3389/fmicb.2015.00358 (PMC4415585; doi:10.3389/fmicb.2015.00358)

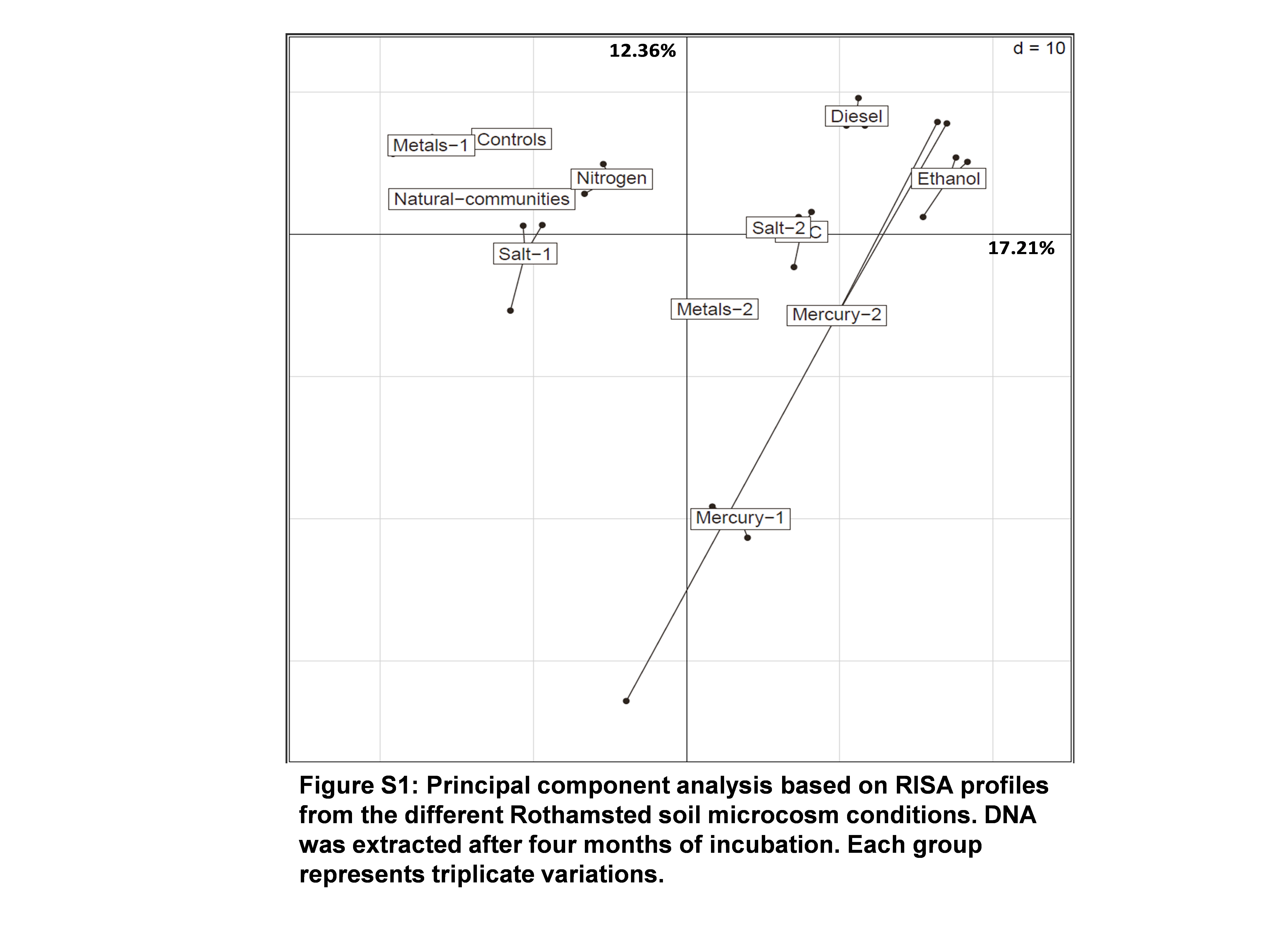

Supplement: Supplementary file 4 [file FigureS1.JPEG]

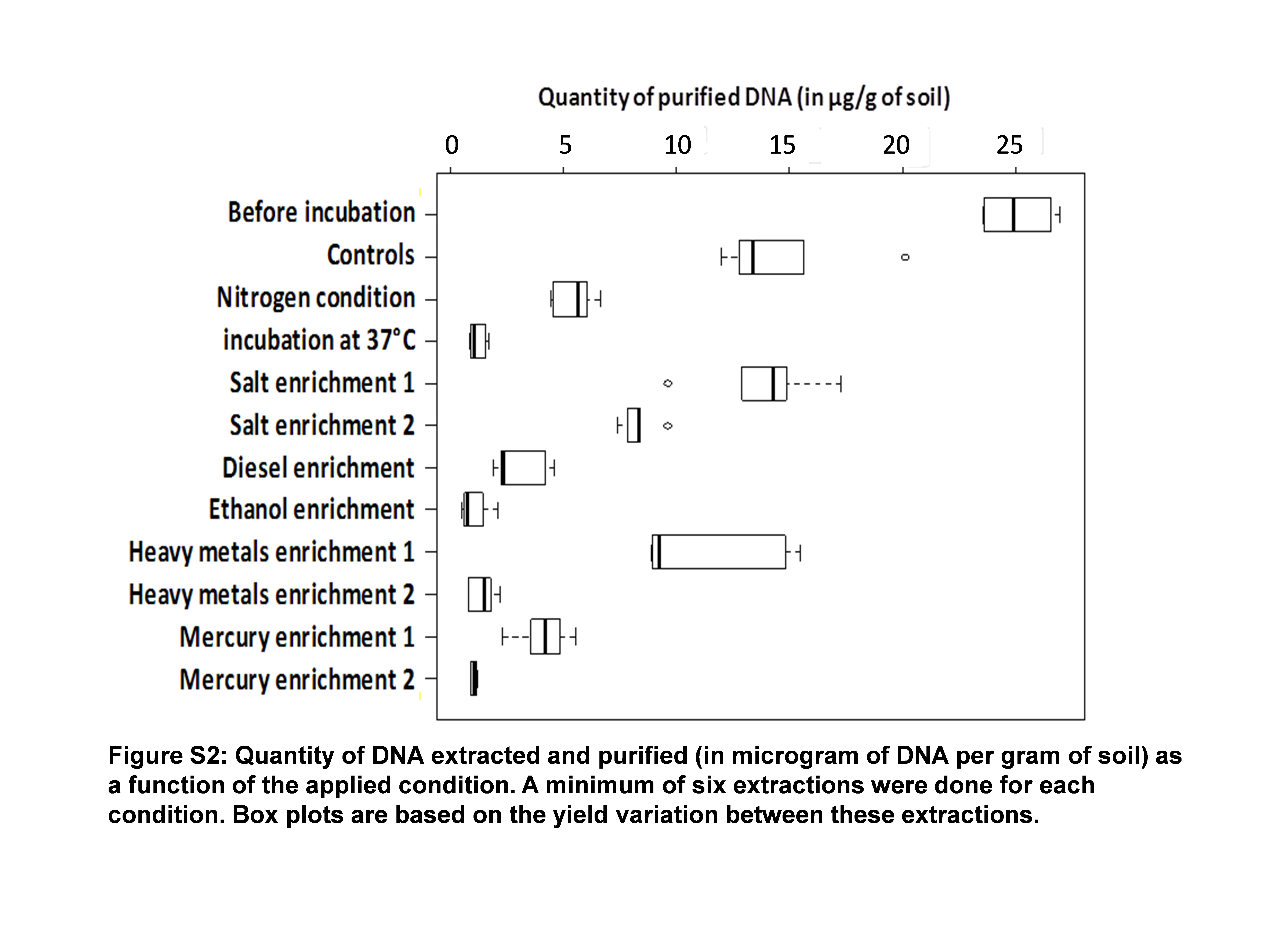

Supplement: Supplementary file 5 [file FigureS2.JPEG]

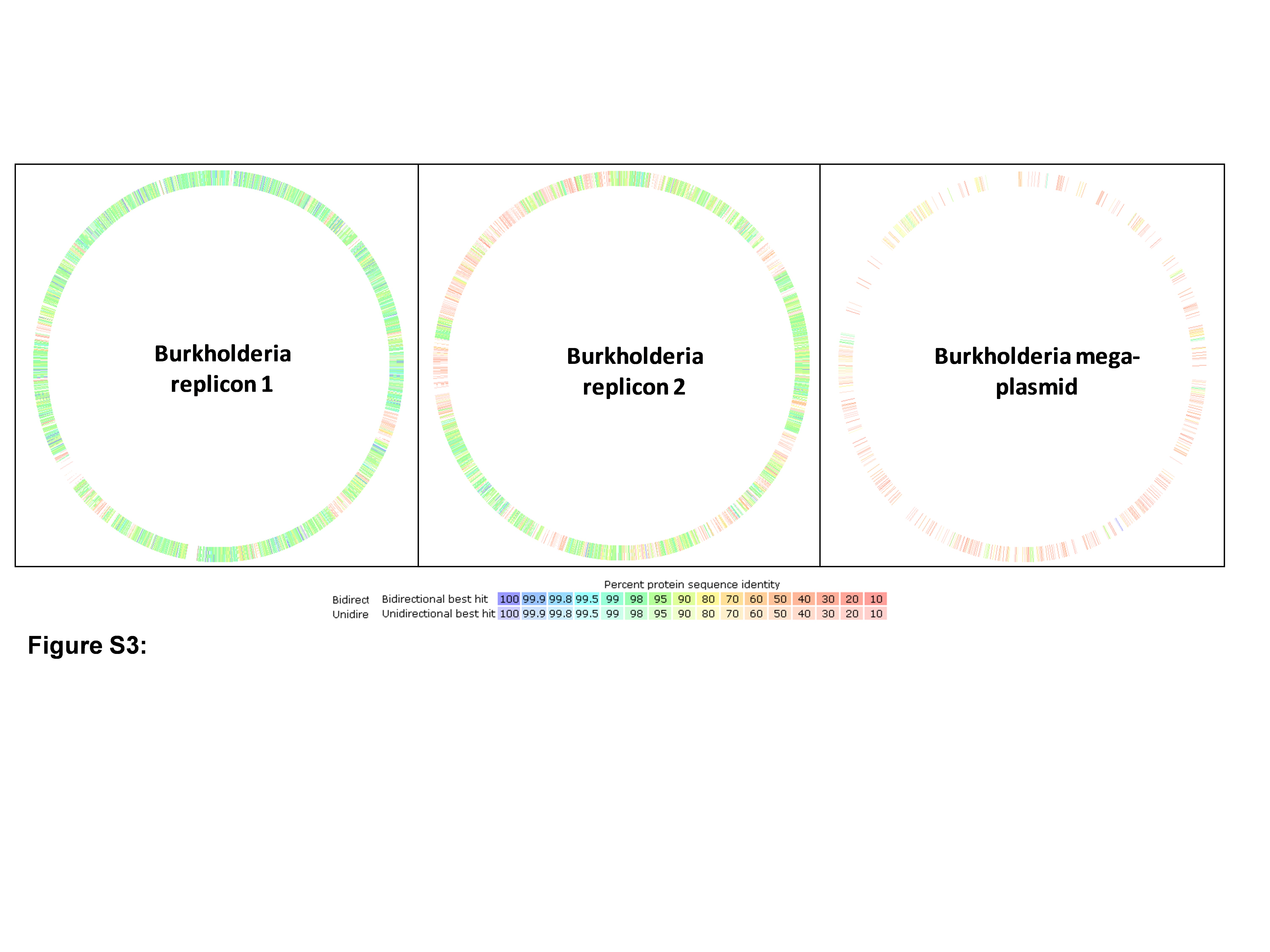

Supplement: Supplementary file 6 [file FigureS3.JPEG]
